# Supplementary material for: A UMLS-based spell checker for natural language processing in vaccine safety
Source: BMC Med Inform Decis Mak. 2007 Feb 12;7:3. doi: 10.1186/1472-6947-7-3 (PMC1805499; doi:10.1186/1472-6947-7-3)
Supplement: Additional file 13 — RAP application directory. Sets up directory for RAP (RDF application for PHP) source code [file 1472-6947-7-3-S13.gz › rap/doc/phpdoc/elementindex_utility.html]

xml version="1.0" encoding="iso-8859-1"?


## [utility] element index

### Package indexes

- model
- dataset
- infModel
- resModel
- ontModel
- syntax
- rdql

All elements
  

c
e
f
g
h
i
k
m
n
o
p
r
s
t
v
w

c

top

createOrd
:   RDFUtil::createOrd() in RdfUtil.php

    Creates ordinal RDF resource out of an integer.

current
:   StatementIterator::current() in StatementIterator.php

    Returns the current statement.

current
:   IterFind::current() in IterFind.php

    Returns the current item

current
:   FindIterator::current() in FindIterator.php

    Returns the current matching statement.

e

top

escapeValue
:   RDFUtil::escapeValue() in RdfUtil.php

    Escapes < > and &

f

top

FindIterator
:   FindIterator::FindIterator() in FindIterator.php

    Constructor

FindIterator
:   FindIterator in FindIterator.php

    Iterator for traversing statements matching a searchpattern.

FindIterator.php
:   FindIterator.php in FindIterator.php

g

top

getCurrentPosition
:   StatementIterator::getCurrentPosition() in StatementIterator.php

    Returns the current position of the iterator.

getLocalName
:   RDFUtil::getLocalName() in RdfUtil.php

    Delivers the Localname (without the namespace prefix) out of the URI of a Resource.

getNamespace
:   RDFUtil::getNamespace() in RdfUtil.php

    Extracts the namespace prefix out of the URI of a Resource.

getOrd
:   RDFUtil::getOrd() in RdfUtil.php

    Converts an ordinal RDF resource to an integer.

getPrefix
:   RDFUtil::getPrefix() in RdfUtil.php

    Short Prefix for known Namespaces by given URI

guessName
:   RDFUtil::guessName() in RdfUtil.php

    Delivers the name out of the URI (without the namespace prefix).

guessNamespace
:   RDFUtil::guessNamespace() in RdfUtil.php

    Extracts the namespace prefix out of a URI.

guessPrefix
:   RDFUtil::guessPrefix() in RdfUtil.php

    Short Prefix for known and/or parsed Namespaces by given URI and Model Uses $default\_prefixes defined in constants.php and getParsedNamespaces() Returns FALSE if no matching prefix is found

h

top

hasNext
:   FindIterator::hasNext() in FindIterator.php

    Returns TRUE if there are more matching statements.

hasNext
:   StatementIterator::hasNext() in StatementIterator.php

    Returns TRUE if there are more statements.

hasPrevious
:   StatementIterator::hasPrevious() in StatementIterator.php

    Returns TRUE if the first statement has not been reached.

i

top

isRDF
:   RDFUtil::isRDF() in RdfUtil.php

    Tests if the URI of a resource belongs to the RDF syntax/model namespace.

IterFind
:   IterFind::IterFind() in IterFind.php

    Constructor.

IterFind
:   IterFind in IterFind.php

    Implementation of a find-iterator which delivers statements or quads.

IterFind.php
:   IterFind.php in IterFind.php

k

top

key
:   IterFind::key() in IterFind.php

    Returns the key of the current item

m

top

moveFirst
:   StatementIterator::moveFirst() in StatementIterator.php

    Moves the pointer to the first statement.

moveLast
:   StatementIterator::moveLast() in StatementIterator.php

    Moves the pointer to the last statement.

moveTo
:   StatementIterator::moveTo() in StatementIterator.php

    Moves the pointer to a specific statement.

n

top

next
:   StatementIterator::next() in StatementIterator.php

    Returns the next statement.

next
:   IterFind::next() in IterFind.php

    Moves Iterator to the next item in the list

next
:   FindIterator::next() in FindIterator.php

    Returns the next matching statement.

o

top

$object
:   FindIterator::$object in FindIterator.php

Object
:   Object in Object.php

    An abstract object.

Object.php
:   Object.php in Object.php

p

top

$predicate
:   FindIterator::$predicate in FindIterator.php

previous
:   StatementIterator::previous() in StatementIterator.php

    Returns the previous statement.

r

top

RDFUtil
:   RDFUtil in RdfUtil.php

    Useful utility methods.

rewind
:   IterFind::rewind() in IterFind.php

    Resets iterator list to start

RdfUtil.php
:   RdfUtil.php in RdfUtil.php

s

top

StatementIterator
:   StatementIterator::StatementIterator() in StatementIterator.php

    Constructor

StatementIterator
:   StatementIterator in StatementIterator.php

    Iterator for traversing models.

StatementIterator.php
:   StatementIterator.php in StatementIterator.php

t

top

toString
:   Object::toString() in Object.php

    Serializes a object into a string

v

top

valid
:   IterFind::valid() in IterFind.php

    Says if there are additional items left in the list

visualizeGraph
:   RDFUtil::visualizeGraph() in RdfUtil.php

    Generates a dot-file for drawing graphical output with the

w

top

writeHTMLTable
:   RDFUtil::writeHTMLTable() in RdfUtil.php

    Prints a MemModel as HTML table.

c
e
f
g
h
i
k
m
n
o
p
r
s
t
v
w
